# How do Informal Caregivers of Seniors’ Tasks Lead to Presenteeism and Absenteeism Behaviors? A Canadian Quantitative Study

**DOI:** 10.3390/ijerph20075392

**Published:** 2023-04-04

**Authors:** Marie-Ève Beauchamp Legault, Denis Chênevert, Francis Maisonneuve, Sari Mansour

**Affiliations:** 1 HEC Montréal, Human Resources Management Department, 3000 Côte-Sainte-Catherine, Montreal, QC H3T 2A7, Canada; 2 School of Administration Sciences, TÉLUQ University, Montréal, QC H2S 3L5, Canada

**Keywords:** informal caregiving, caregiving for seniors, role conflict, conservation of resources theory, presenteeism, absenteeism, family–work conflict, emotional exhaustion

## Abstract

This study extends our knowledge on the role of informal caregivers of seniors and the impact of this role on presenteeism and absenteeism at work. Based on the conservation of resources theory, this article seeks to gain insights into the mechanisms and antecedents of presenteeism and absenteeism among employees who are also informal caregivers of seniors. Specifically, this article argues that family–work conflict and emotional exhaustion mediate the relationship between the informal caregiver’s role, presenteeism, and absenteeism. Quantitative data (questionnaire) from this cross-sectional study were collected from 915 informal caregivers of seniors from 8 Canadian organizations. Structural equation modelling (SEM) was undertaken using IBM SPSS AMOS 28.0 to test all hypotheses. Informal caregivers of seniors who need to coordinate and organize healthcare are at a higher risk of experiencing family–work conflict. Family–work conflict experienced by informal caregivers subsequently leads to emotional exhaustion, presenteeism, and absenteeism. Because informal caregiving of seniors is likely to increase in coming years for many workers, organizations must be aware of the possible consequences of this role on work productivity. This study shows that not all tasks of informal caregivers of older adults lead to presenteeism and absenteeism through family–work conflict and emotional exhaustion. This study is innovative because, to our knowledge, no study of informal caregivers of older adults has examined the effect of different tasks in this role on presenteeism and absenteeism.

## 1. Introduction

In many countries around the world, the accelerated ageing of the population is a widespread demographic change. According to the World Health Organization [1], the population aged 60 and over will double between the 2000s and the 2050s. One in six people worldwide will be over the age of 65 by 2050, compared to one in eleven in 2019 [2]. Canada is no exception, with the proportion of people aged 65 and over expected to reach 24% by 2036 [3]. Various factors explain these changes within the Canadian population, including the decline in birth rate, increase in life expectancy after age 65, and the ageing of the baby-boom cohort [4].

From an organizational point of view, the ageing of the population is not without consequences, as living longer with chronic disabling diseases requires more support [5]. Informal caregivers can help aging people, reducing demand of healthcare systems. Informal caregivers are family, neighbors, or friends who provide unpaid assistance or help to someone with diminishing physical and/or cognitive abilities and/or a chronic life-limiting illness with activities of daily living and/or medical tasks [6,7]. The number of informal caregivers of seniors (i.e., caring for older people that are 65 and older) is estimated to be 32.4 million in the United States [7], more than 8 million in Canada [8], and close to 1.5 million in Quebec (a Canadian province), 56% of whom are also workers [9]. Generally, women are more likely to be informal caregivers than men [10,11]. The current situation indicates that there will be an increased presence of employees who will have to help an ageing person in the coming years. Many workers already have to conciliate their work with family responsibilities, but many must, or will soon have to, care for a senior, thus becoming informal caregivers.

In recent years, government measures to assist caregivers of seniors have been put in place to help alleviate the effects of this role for these individuals. For example, in Canada, benefits and tax credits are available for people who have to care for a senior who is ill, injured, or receiving end-of-life care. However, these benefits are only available upon specific criteria and for a limited period. These benefits do not necessarily cover the entire period during which the worker is required to be an informal caregiver for an older adult [12]. Non-profit organizations (NPOs) have also emerged with a mission to improve the quality of life for informal caregivers of seniors in Canada. However, these resources are often not sufficient to provide financial assistance to informal caregivers of seniors who must continue to work to support themselves and their families.

The burden of caregiving can significantly impact caregivers and their work productivity, and the consequences of loss of work productivity due to presenteeism and absenteeism are more critical for informal caregivers than for non-caregivers [5]. Presenteeism refers to going to work despite feeling unhealthy (physically or psychologically) or experiencing other events that might typically compel absence [13,14,15], whereas absenteeism refers to an employee’s habitual absence from work [16]. This finding is alarming for organizations, given that there will be an increasing number of employees in caregiving situations in coming years. However, the antecedents of absenteeism and presenteeism among informal caregivers of seniors need to be better documented in the literature [17]. Our study attempts to fill this research gap and deepen our understanding of the mechanisms leading to these behaviors. This study may help organizations better target their intervention with employees who are also caregivers.

This article, therefore, has two main objectives. First, it breaks new ground by dissecting the burden of informal caregivers’ tasks. This approach enables us to better understand the relationship between the informal caregiving role and the loss of work productivity. This study innovates by measuring the impact of informal caregiver duties (e.g., housework, emotional support, coordination of healthcare) on presenteeism and absenteeism. Studies on informal caregiving have mainly focused on the effect of the number of hours invested in this role on some work behaviors, regardless of the type of task [18]. The current literature does not provide information regarding which informal caregiving tasks could be considered more “resource-depleting”. Second, as informal caregiving tasks drain resources (e.g., time, energy, money), this may lead to family–work conflict, defined as the difficulty for a worker to balance family and work roles [19]. According to the conservation of resource theory (COR) [20], the initial loss of resources can lead to future losses, resulting in emotional exhaustion, defined as a lack of energy, and a feeling that one’s emotional resources are depleted [21]. Based on the COR theory, this article argues that family–work conflict and emotional exhaustion mediate the relationship between the caregiver’s role (independent variables), presenteeism (dependent variable 1), or absenteeism (dependent variable 2). 

The following sections begin by presenting our theoretical framework and the development of the research hypotheses. Next, after presenting the methodology and testing the hypotheses, the main results are discussed. This article then highlights this study’s theoretical and practical implications and limitations and identifies interesting directions for future research.

## 2. Theoretical Framework and Hypotheses

### 2.1. The Burden of Informal Caregiving

Many informal caregivers must balance their family responsibilities with work. Depending on their age and financial needs, some caregivers may not yet be ready to retire or have other responsibilities requiring them to work simultaneously (such as caring for their children). On this subject, a report by the AARP Public Policy Institute states that 69% of informal caregivers reported adjusting their work performance by arriving late, leaving work early, changing, or stopping work to balance this role with their caregiving role [22]. In Canada, more than half of informal caregivers of seniors also work more than 30 h per week [9], which can increase their perception of role conflict. According to Greenhaus and Beutell [19], role conflict between family and work occurs when (a) the time, (b) the demands, and/or (c) the specific behaviors spent on the demands of one role make it more challenging to meet the demands of the other role. A report shows that 70% of working caregivers are affected by problems related to incompatibility of their roles [23]. For informal caregivers of seniors, the time spent in their caregiving role can undoubtedly make it more difficult to satisfy their work role and create constraints. 

Informal caregivers of seniors take on multiple roles in the life of the person they care for. It is possible to break down the tasks of informal caregivers of seniors into six different categories as follows: (1) housework; (2) personal care; (3) social and emotional support; (4) health and medical care; (5) organizing and coordinating healthcare; and (6) acting as a substitute (see Table 1 for more details on each task) [24,25].

The role of informal caregivers significantly increases individuals’ stress levels due to the time commitment and perceived burden of the task. Indeed, informal caregivers are more affected by anxiety, depression, worry, and loneliness than non-caregivers [26]. Informal caregivers often must make lifestyle changes that decrease “restorative activities”, such as leisure and contact with friends and family, to fulfill their roles [27]. 

### 2.2. The Relationship between Informal Caregiving Role and Family–Work Conflict

**Figure 1:**
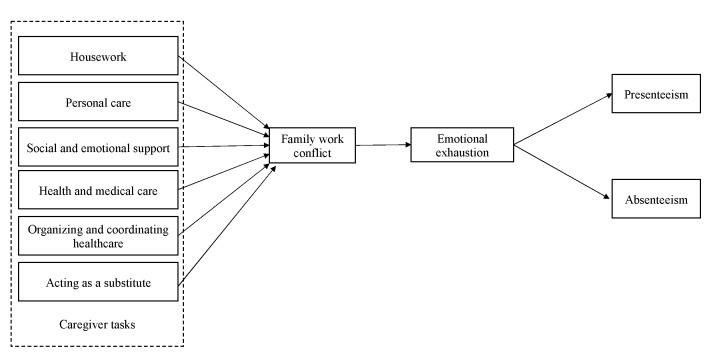
The theoretical model.

Furthermore, the informal caregiving role consumes energy and attention that cannot be reinvested in work. In line with the COR theory [20], family demands (i.e., informal caregiving tasks) drain resources from the worker who must combine both roles (and often many other roles). The COR theory proposes that people strive to retain, protect, and build resources and that the potential or actual loss of these resources is a threat to them [20]. For informal caregivers, some aspects of their role can threaten and destabilize their resources. As many informal caregivers must balance their family responsibilities with work, this role creates more family–work conflict [28]. Research on the effects of informal caregiving roles focuses primarily on the number of hours invested in the role [18]. Our theoretical model, presented in Figure 1, considers the effects of different roles that caregivers of older adults may perform (i.e., housework, personal care, social and emotional support, health and medical care, organizing and coordinating healthcare, acting as a substitute) on family–work conflict. Consistent with the COR theory, we wish to explore how involvement in different informal caregiving tasks are perceived as distinct resource threats which influence family–work conflict. We therefore postulate that the number of hours informal caregivers invest in different caregiving tasks has a positive relationship with family–work conflict.

**Hypothesis** **1.** *The number of hours spent by the informal caregivers on (a) housework tasks; (b) personal care tasks; (c) social and emotional tasks; (d) health and medical care tasks; (e) organizing and coordinating healthcare tasks; and (e) acting as substitute tasks will be positively associated with family–work conflict*.

### 2.3. The Relationship between Family–Work Conflict and Emotional Exhaustion

Family–work conflict can significantly impact informal caregivers’ mental and physical health by increasing emotional exhaustion [29,30]. Emotional exhaustion is a lack of energy and a feeling that one’s emotional resources are depleted [21]. Prior research has linked work–family conflict with poorer psychological health [31,32]. Based on the COR theory [20], the caregiving role involves meeting many additional demands, despite the informal caregiver’s everyday work or personal activities, increasing the potential for resource loss. Due to the multiplicity of their roles, informal caregivers may not have enough time or energy to complete their work tasks and duties associated with their informal caregiving role. Emotional exhaustion is more likely to occur when the individual feels that they do not have enough time to do what they need to do, when there is a high emotional demand for their role, or when there is role conflict or role ambiguity [33,34]. As a second hypothesis, we state that emotional exhaustion occurs because of a lack of resources, as work–family conflict would not allow any gain at this level. Thus, we propose the following hypothesis:

**Hypothesis** **2.** *Family–work conflict will be positively associated with emotional exhaustion.*

Moreover, the COR theory discusses the corollary of the loss spiral cycle [20,35]. This corollary state that loss of resources leads to more stress and further loss of resources [35]. Because of high demands in their family domain, working informal caregivers are more likely to experience losses of resources in other life areas. These losses of resources could occur in their work sphere and exacerbate family–work conflict. These losses then increase the caregiver’s stress spiral, resulting in emotional exhaustion. We hypothesize that family–work conflict mediates the relationship between the number of hours spent on each task by the informal caregiver and their emotional exhaustion state.

**Hypothesis** **3.** *Family–work conflict will mediate the positive association between the number of hours spent on each task by the informal caregiver and their emotional exhaustion.*

### 2.4. The Relationship between Emotional Exhaustion, Presenteeism, and Absenteeism

Presenteeism and absenteeism are more prevalent among working informal caregivers as these behaviors can decrease work productivity from 22.9% to 27.4% [5,36]. Studies have shown that emotionally exhausted informal caregivers are more prone to presenteeism [37,38]. Some symptoms associated with emotional exhaustion (e.g., apathy, stress) can interfere with workflow by making them unable to perform as usual, thus increasing presenteeism [38]. Emotionally exhausted workers are also prone to more absenteeism [39,40,41]. For various reasons, some emotionally exhausted informal caregivers may take time off work instead of showing up for work. Again, according to the COR theory, for some informal caregivers, taking time off work could be another way to avoid further loss of resources. Indeed, the fourth corollary of the Conservation of Resource Theory states that a lack of resources could lead to defensive attempts to conserve remaining resources [20,35]. Emotionally exhausted informal caregivers may also be more prone to health concerns related to their emotional state, increasing their propensity to take time off work [39]. Thus, we suggest that emotional exhaustion could lead to more absenteeism or presenteeism behaviors for informal caregivers of older adults.

**Hypothesis** **4.** *Emotional exhaustion will be positively associated with (a) absenteeism and (b) presenteeism*.

As previously stated, the corollary of the loss spiral cycle [35] causes resource loss (or gain) to spiral. This corollary state means that each loss will result in resource depletion that the subsequent loss will exacerbate, and so on, resulting in a cascade of resource loss. As a final hypothesis, we propose that emotional exhaustion mediates the positive association between family–work conflict and (a) absenteeism and (b) presenteeism. The emotional exhaustion experienced by informal caregivers experiencing family–work conflict would subsequently increase the risk of absenteeism and presenteeism of these individuals.

**Hypothesis** **5.** *Emotional exhaustion will mediate the positive association between family–work conflict and (a) absenteeism and (b) presenteeism.*

## 3. Materials and Methods

### 3.1. Data Collection and Sample

The sample for this study was drawn from a sample of a larger research project that examined the impact of sociodemographic changes characterized by an aging population and increasingly complex family responsibilities in Canada in 2018. The data were collected using an online questionnaire from 2600 employees of eight Canadian organizations (automotive industry, manufacturing industry, insurance sector, research and development, health and social services, municipal sector). With convenience sampling, a dozen organizations were contacted by the research team according to their sector of activity so that the sample would represent different sectors of the economy. Then, the organizations were selected after demonstrating their interest in participating in this study. All employees of these organizations were invited to complete the questionnaire. A final sample of 1393 questionnaires was acquired, which were completed with a response rate of 53.5%. Approval by the Research Ethics Board from the university was obtained for the project before the study started, and informed consent was obtained from participants before completing the surveys.

Because this study focuses on informal caregivers of people over 65, only questionnaires from those who answered this question were included in the analysis. Of the 1393 participants, 961 indicated they were informal caregivers of people over 65. This subsample represented 69% of the original database. After suppressing outliers and missing values, the final sample comprised 915 informal working caregivers. 

### 3.2. Descriptive Statistics and Correlations

Table 2 presents the descriptive statistics and correlations for the study variables. 

Regarding descriptive statistics of the informal caregivers’ sample, women comprised 52.1% of the sample (*n* = 477) and men 47.9% (*n* = 438). The respondents’ ages varied between 17 and 73 years, with an average age of 43,7 years. A total of 28 % of informal caregivers had a high school degree, 17.2% had a diploma of college study, 7.2% had an undergraduate certificate, 21.4% had a bachelor’s degree, 16.7% had a master’s degree, and 4.4% had a doctorate. The number of dependents (children, spouses, etc.) varied significantly across the sample, with a minimum of 0 dependents and a maximum of 8. On average, respondents had 1.67 dependents. Informal caregivers within our sample spent an average of 38 h at work per week and 14.5 h per week caring for their children. Time spent on informal caregiving tasks varied considerably in the sample, ranging from 0 to 60 h per month, depending on the task, with a total average of 2.21 h per task per month. Table 3 summarizes respondents’ average hours per month on each task.

### 3.3. Measures 

As the study was conducted in French and English (95% and 5% of the respondents, respectively), all instruments used in French were translated from English using a standard translation–back translation procedure [42]. Any translation discrepancies were resolved through a brief discussion among the translators. The questionnaires were also pretested among 25 respondents in French and English to ensure all items were clearly understood. The following section presents the instruments used to measure the constructs.

Independent variable
Roles of informal senior caregivers

A measurement scale was developed to assess the informal caregivers’ roles based on information drawn from research reports and government sources [24,25]. Those sources described the tasks that informal caregivers of seniors must usually perform daily. For each of the tasks, respondents needed to estimate, on a scale ranging from 0 h to 10 h or more, how many hours in the past month were spent on each of the following caregiving responsibilities: housework; personal care; social and emotional support; health and medical care; healthcare organization and coordination; and acting as a substitute.

Dependent variables
Presenteeism

Presenteeism was measured using the validated Stanford presenteeism scale (six-item scale), as recommended by Turpin et al. [43]. The caregivers were asked to rate, on a scale of 1 to 4 (from “almost never” to “always”), their presenteeism behaviors at work (e.g., “I had problems concentrating”, “I had to work at a slower pace”) from the last two weeks due to various health problems (e.g., fatigue, stress, exhaustion, physical or psychological problems). Regarding the psychometric properties of this scale, this instrument’s internal consistency was ∂ = 0.84.

Absenteeism

Absenteeism was measured with three questions, asking the respondents to report in the last three months how many days (0 days to 10 days and more) they were unable to report for work or to perform their usual work because of health problems, responsibilities to their children, or responsibilities to a senior (age 65 and over). An overall score of these three absenteeism types was created to measure each participant’s average absenteeism for the last three months.

Mediating variables
Family–work conflict

Family–work conflict was measured with three items of the “Work and Family Conflict Scale” subscale measuring family-to-work conflict (∂ = 0.77) [44]. Caregivers were asked to rank on a seven-point Likert scale ranging from “strongly disagree” to “strongly agree” items related to family–work conflict (e.g., “My family and personal responsibilities are so great that it interferes with my job” “My family and personal life takes up time that I would like to spend at work”).

Emotional exhaustion

Items linked to emotional exhaustion were taken from the Maslach Burnout Inventory-Human Services Survey (MBI-HSS) [21]. Five items were used to assess emotional exhaustion. A sample item for emotional exhaustion is “I feel burned out from my work.”. The internal consistency reliability for this scale was ∂ = 0.91.

Control variables:

We controlled for some sociodemographic variables that could have effects on the variables of our model, such as (1) age, (2) gender, and (3) the number of dependents, such as children or spouses, following previous research and recommendations on informal caregiver research [38,45].

## 4. Analysis

### 4.1. Preliminary Analyses

Before proceeding with the hypothesis analyses, preliminary analyses were conducted to verify the accuracy of data entries, missing data, or outliers. Assumptions of multivariate statistics were also analyzed and determined satisfactorily. For normality, the data showed no skewness or kurtosis issues. For multicollinearity, for all variables, the VIF was less than 5 [46].

### 4.2. Data-Analysis Strategy

Structural equation modelling (SEM) was undertaken using IBM SPSS AMOS 28.0 to test all hypotheses. First, the measurement model was estimated using confirmatory factor analysis (CFA) before considering the structural model, as recommended by Anderson and Gerbings’s [47] two-step approach. Maximum likelihood estimation was used because the data were normally distributed. Gaskin’s indirect effect estimand for Amos was used to test the mediations. This estimand is a macro for Amos using a bootstrapping procedure to test the indirect effect [48]. For bootstrapping, 5000 cases and a 95% confidence interval were utilized. All betas presented in the result section were standardized.

### 4.3. Measurement Model

#### 4.3.1. CFA

We proceeded to confirmatory factor analysis for the three latent constructs of this study according to Anderson and Gerbing’s approach [47]. Many commonly used fit indices were used to assess the confirmatory factor analysis, including the following: the comparative fit index (CFI), for which values of 0.90 or higher indicate a good fit; the root mean square error of approximation (RMSEA), for which values lower or equal to 0.08 indicate a good fit; and the standardized root mean residual (SRMR), for which values of 0.09 or lower indicate a good fit [49]. Overall, the hypothesized model was composed of three latent factors (family–work conflict, emotional exhaustion, and presenteeism) and yielded a good fit to our data (X2 (30) 88.93 = *p* < 0.000, CFI = 0.987, RMSEA = 0.047, SRMR = 0.042). 

#### 4.3.2. Common Method Bias

As the data were all self-reported, common method bias could affect the results. A common method bias test was conducted using a common latent factor [50,51]. The common latent factor (CLF) captures our model’s common variance among all observed variables. We compared the unconstrained common method factor model with the fully constrained common method factor model. The chi-squared test was significant (*p* = 0.000). This result means that there was significant shared variance with our measurement method, so we decided to keep the CLF as we moved into structural model analyses [50,51]. 

#### 4.3.3. Control Variables

Regarding the control variables of family–work conflict, age was negatively associated with family–work conflict (ß = −0.077, *p* = 0.017). The older the informal caregiver was, the less they experienced family–work conflict. Furthermore, the number of dependents the caregiver had was positively associated with family–work conflict (ß = 0.177, *p* = 0.01); the more people the caregivers had in their care, the more this conflict was felt. Additionally, gender (coded as man = 1, woman = 2) had a significant impact on average absenteeism and presenteeism behaviors: women tended to have more absenteeism (ß = 0.214, *p* = 0.01) and more presenteeism (ß = 0.065, *p* = 0.04) behaviors than men. In addition, the number of dependents significantly impacted absenteeism (ß = 0.133, *p* = 0.01); the more dependents the caregiver had (e.g., children, spouse, etc.), the more the caregiver was absent. 

## 5. Results

**Figure 2:**
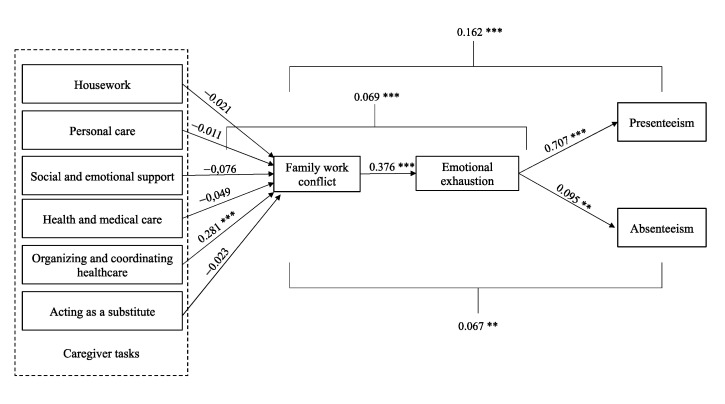
The final structural model with standardized path coefficients ** *p* < 0.01, *** *p* < 0.001.

As shown in Figure 2, hypothesis 1 (a), 1 (b), 1 (c), 1 (d), and 1(f) proposed, respectively, that housework, personal care, social and emotional support, health and medical care, and acting as substitute tasks were positively related to family–work conflict for informal caregivers. However, no significant relationship was found for those hypotheses (H1a: *p* = 0.618, H1b: *p* = 0.815, H1c: *p* = 0.062, H1d: *p* = 0.363, H1f: *p* = 0.645). Hypothesis 1 (e) proposed that organization and coordination of healthcare tasks for informal caregivers were positively related to family–work conflict. The results show a significant positive relationship (ß = 0.281, *p* = 0.001), thus supporting hypothesis 1 (e).

Hypothesis 2 suggests that family–work conflict is positively associated with emotional exhaustion. The analysis results show that work–family conflict is positively associated with emotional exhaustion (ß = 0.376, *p* = 0.001), thus confirming hypothesis 2.

Hypothesis 3 suggested that family–work conflict mediates the positive association between the number of hours spent on each task by the informal caregiver and their emotional exhaustion. Because only healthcare coordination appeared to have a positive relationship with work–family conflict regarding hypothesis 1, this hypothesis was tested using only the healthcare coordination task as the independent variable. Mediation analysis confirms that family–work conflict positively mediates the relationship between the number of hours spent participating in healthcare coordination and emotional exhaustion for informal caregivers (ß = 0.069, *p* = 0.001; LLCI: 0.033, ULCI: 0.102). Therefore, hypothesis 3 is supported.

Hypothesis 4 stated that emotional exhaustion is positively associated with (a) absenteeism and (b) presenteeism. Regarding the positive association between emotional exhaustion and (a) absenteeism and (b) presenteeism, the analyses show a significant association between absenteeism (ß = 0.095 *p* = 0.002) and presenteeism (ß = 0.704 *p*= 0.001). Hypotheses 4 (a) and 4 (b) are supported. 

Finally, hypothesis 5 stated that emotional exhaustion mediates the positive association between family–work conflict and a) absenteeism and b) presenteeism. Regarding the positive mediation of emotional exhaustion on family–work conflict and absenteeism, the analysis shows a significant relationship (ß = 0.067 *p* = 0.002; LLCI: 0.020, ULCI: 0.117). Finally, the analyses also show a significant association for the positive mediation of emotional exhaustion between family–work conflict and presenteeism (ß = 0.162 *p* = 0.001, LLCI: 0.130 ULCI: 0.195). Thus, hypotheses 5 (a) and 5 (b) are supported.

### Supplementary Analysis 

As an additional analysis, we tested whether the serial mediation worked with emotional exhaustion as a first mediator and family–work conflict as a second mediator to confirm the order of the variables in our model. For this model, the fit indices were unsatisfactory [49] (X2 (20) 760.388 = *p* < 0.000, CFI = 0.827, RMSEA = 0.201 = *p* > 0.00, SRMR = 0.1202). This analysis confirms the sequence of the mediators in our initial model. This supplementary analysis is available upon request.

## 6. Discussion

Based on the COR theory [20], the present paper aimed to understand the mechanisms and antecedents of presenteeism and absenteeism among informal caregivers’ tasks through family–work conflict and emotional exhaustion. This study sought out which tasks among seniors’ informal caregivers were most likely to lead to presenteeism and absenteeism behaviors. 

First, our results show that, for informal caregivers of seniors, organizing and coordinating healthcare is positively related to family–work conflict. Informal caregivers of seniors who are required to perform this role are at greater risk of experiencing family–work conflict; this task appears to drain more resources, such as time and energy, favorizing conflict. Interestingly, no other tasks were significantly related to family–work conflict in our study. It is also interesting to note that this task did not take up the most hours for informal caregivers in our sample. On average, participants spent 1.54 h on healthcare organization and coordination in the past month, with a standard deviation of 2.21 h. However, our results show that coordinating and organizing healthcare does not seem to be an easy task for informal caregivers. Having to search for information, communicating with different actors in the healthcare system, or making medical appointments appears to have a significant effect on family–work conflict among informal caregivers. The complexity of navigating the healthcare system may explain why informal caregivers feel such pressure [52]. Schulz and Thompkins [25] note that healthcare coordination can be a significant challenge for informal caregivers of seniors. Indeed, not all informal caregivers are comfortable with health literacy. Understanding the care and service options available to seniors is not always straightforward and can be a puzzle for some, taking over a significant amount of resources [52]. Informal caregivers probably also have to coordinate healthcare while at work, such as scheduling appointments or talking to professionals, thus increasing family–work conflict. 

In our study, the strain caused by family–work conflict may play an important role in the relationship with emotional exhaustion for informal caregivers of seniors. Consistent with previous empirical findings [29,30,33] and the COR theory’s loss spiral cycle corollary [20,35], our results show that family–work conflict is positively associated with emotional exhaustion and that it positively mediates the relationship between the informal caregiver’s tasks (i.e., coordination of healthcare) and emotional exhaustion. Due to various demands from family and work domains, the informal caregiver’s family–work conflict increases the propensity for informal caregivers to experience emotional exhaustion. 

Moreover, our results show that emotional exhaustion is positively related to presenteeism and absenteeism and mediates the relationship between family–work conflict, presenteeism, and absenteeism. First, regarding the effects on presenteeism, these results are consistent with studies showing that emotional exhaustion can make workers less able to work, thereby increasing their presenteeism behaviors [53,54]. Recently, Karanika-Murray and Biron [55] described presenteeism as an adaptive behavior, allowing the individual to balance health constraints with the performance required at work, especially when health problems are not contagious (e.g., by being emotionally exhausted). According to this perspective, presenteeism can be therapeutic (i.e., taking refuge in work without being productive), functional (i.e., having a good balance between health constraints and performance), dysfunctional (i.e., having poor work performance and poor health), or overachieving (i.e., when performing at work comes at the expense of health) [55]. For emotionally exhausted caregivers, showing up to work may be a form of therapeutic presenteeism by allowing them to meet basic psychological needs, such as affiliation, autonomy, or competence [56]. This behavior limits the loss of resources experienced by working informal caregivers. Indeed, by showing up to work, some caregivers may seek comfort from their colleagues or a break from their informal caregiving role. An organizational climate may play an important role in the type of presenteeism of some informal caregivers, as work can become a source of support. Indeed, the motivations and consequences of presenteeism in a caregiver-friendly workplace may not be the same for an informal caregiver working in an organization that does not offer such a work climate. Second, for absenteeism, our results are consistent with various empirical studies showing a positive relationship between emotional exhaustion and absenteeism [39,40,57]. Indeed, emotional exhaustion increases sickness absenteeism [57]. Some informal caregivers of older adults may decide to take time off work rather than go to work because of emotional exhaustion, thus explaining why, in this study, emotional exhaustion was also related to absenteeism. The decision for informal caregivers to take time off work or go to work despite this exhaustion could be explained by the presence (or absence) of perceived work pressure. Indeed, some informal caregivers may feel pressure to come to work despite everything, while others may have more flexibility and can easily be away from work. However, further studies are necessary to understand this phenomenon.

## 7. Theoretical and Practical Implications

This research has interesting theoretical and practical implications. Theoretically, this article provides a better understanding of the effect of different informal caregivers’ roles on presenteeism and absenteeism via family–work conflict and emotional exhaustion. The results of our study add to the conservation of resources theory and the loss spiral cycle corollary [20,35]. Specifically, this study advances our understanding of the corollary of the loss spiral cycle leading to presenteeism and absenteeism behaviors with a tangible representation of this phenomenon for informal caregivers. This study shows that family–work conflict felt by informal caregivers that must coordinate healthcare accentuates a loss of resources. This loss of resources then leads to emotional exhaustion, another loss for informal caregivers. Subsequently, the sequence of family–work conflict and emotional exhaustion would lead to a loss of work productivity among caregivers, resulting in presenteeism and absenteeism behaviors. In short, the loss of resources created by coordinating and organizing healthcare for informal caregivers creates a cascade of loss of resources (loss spiral cycle), leading to a loss in work productivity. In addition, this study also addresses the need to better understand the phenomenon of presenteeism and absenteeism among informal caregivers of seniors [17].

Practically, our study can help organizations understand the effect of various informal caregiving roles on presenteeism and absenteeism through family–work conflict and emotional exhaustion. Indeed, this mechanism is important to understand, as presenteeism and absenteeism can significantly impact employee productivity. Because the amount of informal caregiving of seniors is likely to increase in the coming years, organizations must be aware of the possible consequences of this role on work productivity. This study shows that not all caregiving hours affect informal caregivers similarly. Our study shows that coordinating and organizing healthcare is more problematic for informal caregivers. As a result, organizational and government interventions should pay more attention to the challenges of healthcare coordination for informal caregivers. More concretely, governments need to facilitate the coordination and organization of healthcare for informal caregivers by increasing available resources, using telemedicine platforms, for example, or developing tools that can help coordinate the healthcare pathway of people who are losing their autonomy. In addition, a favorable work environment and corporate culture would allow policies and practices to be put in place to balance the demands of the informal caregiving role with those of the work role. Some programs, such as employee assistance programs, in collaboration with NPOs or government programs, could provide information and training sessions to guide informal caregivers through the healthcare system. In short, our study demonstrates the importance of organizations and the government in paying particular attention to the services and supports available to informal caregivers.

## 8. Limitations

This study has some limitations that need to be addressed. First, the data for this study were collected using a single measurement time. This data collection method allowed us to measure many variables without attrition risk. However, with such a methodology, we cannot establish a causal link between the variables. Future studies with longitudinal data collection would be preferable to detect changes over time in the variables. Longitudinal studies would make it possible to detect changes in the mediating variables over time. Indeed, mediators’ measurement with cross-sectional studies is not optimal because mediations are “causal processes that unfold over time” [58] (p. 23). Longitudinal studies would also increase the external validity of the results. Second, identifying organizations with convenience sampling has some limitations [59]. Because the sample is not chosen through random selection, the generalization of the results is more complex [59]. However, all employees of the participating organizations could decide whether to participate in the study, thus adding a notion of randomization to our sample. Third, all the data collected in this study were self-reported. Some caregivers may have underestimated or overestimated the time allocated to specific tasks in their caregiving role. This underestimation or overestimation may have had an impact on the results. For example, some participants may have underestimated the time allocated to specific tasks, which could explain why, in our model, only one informal caregiving task has a significant positive relationship with family–work conflict. Future studies should use a more objective way to measure the time allocated to informal caregiving tasks. For example, for over two weeks, informal caregivers of seniors might be asked to complete a daily questionnaire measuring their time on each task. This technique would be more accurate than asking them to estimate how much time they allocated to each task in the past month. In addition, because all data were self-reported, we experienced a common method bias problem, although this deficiency was addressed before data analysis. Diversity in data sources are necessary for future studies to prevent such a problem. Fourth, online questionnaires can have limitations in terms of sample selection, response, and non-response bias. Technological problems can also occur during the completion of the surveys, limiting the quality of the responses obtained [60]. However, we believe we were able to minimize the potential bias of this data collection method in this study. Using online questionnaires for this study allowed us to reach a large sample of informal caregivers.

## 9. Future Research Directions

Finally, as previously mentioned, a longitudinal study would be interesting for future research directions to establish the causal relationship between the variables. Moreover, a qualitative approach would be more than relevant. Indeed, this would provide a better understanding of some of the relationships in this study. This approach may provide insight into why coordinating healthcare tasks are more resource depleting for informal caregivers of seniors than the tasks. A qualitative approach could also help to understand how and why these roles lead to family–work conflict among informal caregivers of seniors. In addition, measuring the perceived intensity of caregiving as a control variable is relevant. Indeed, the perceived intensity of this role could further exacerbate the relationship between some variables. It would also be interesting to add the effect of physical distance between the caregiver and the elderly person being cared for as a variable in a future study. Indeed, whether or not the caregiver lives close to the person in need could influence some variables differently. Furthermore, an experimental approach could be interesting when studying the informal caregiving burden. Indeed, as highlighted in the practical implications, it would be helpful for informal senior caregivers to access a telemedicine platform to facilitate healthcare coordination. An experimental study could measure the effect of using this application on exhaustion among informal caregivers of seniors. Finally, to further our understanding of presenteeism and absenteeism among working informal caregivers, future studies should look at the effect of certain aspects of work (e.g., ease of replacement, organizational culture) on these two constructs. 

## 10. Conclusions

Based on the conservation of resources theory, this cross-sectional quantitative study aimed to extend knowledge on the role of informal caregivers of seniors and the impact of this role on presenteeism and absenteeism at work. This study shows that not all tasks of informal caregivers of older adults lead to presenteeism and absenteeism through family–work conflict and emotional exhaustion. Informal caregivers of seniors who need to coordinate and organize healthcare are at higher risk of experiencing family–work conflict. Family–work conflict subsequently leads to emotional exhaustion, presenteeism, and absenteeism behaviors among informal caregivers. Because there will be an increase in the number of workers who will also be informal caregivers to seniors in the future, resources from the government and organizations must be put in place to reduce the impact of this role on the work performance of these individuals.

## Figures and Tables

**Table 1:** The different tasks that informal caregivers of seniors can do and their definitions.

Task	Definition
Housework	Doing laundry, meals, house maintenance, etc.
Personal care	Bathing, toileting, dressing, feeding, supervising the elderly, and helping the person move around.
Social and Emotional Support	Providing presence, talking with the person, organizing and participating in leisure activities, managing family conflicts, problem-solving, and managing emotions.
Health and Medical Care	Encouraging healthy lifestyles, promoting adherence to treatment, managing and administering medications, using medical equipment, preparing meals according to a specific diet, responding to emergencies, and providing care.
Organizing and coordinating healthcare	Seeking information, promoting understanding, communicating with healthcare professionals, coordinating the various stakeholders, making appointments, renewing prescriptions, negotiating with insurance companies, etc.
Acting as a substitute	Dealing with legal and financial issues, managing personal assets, and participating in treatment planning and decisions.

**Table 2:** Descriptive statistics and correlation *.

Variable	M	SD	1	2	3	4	5	6	7	8	9	10	11	12	13
1. Gender	-	-	-												
2. Age	43.80	11.09	0.141 **	1											
3. Number of dependents	1.67	1.41	−0.058 *	−0.025	1										
4. Housework tasks	2.98	3.39	−0.039	0.144 **	−0.007	1									
5. Personal care tasks	1.69	2.59	−0.013	0.123 **	0.019	0.614 **	1								
6. Social and emotional support tasks	3.75	3.60	0.089 **	0.096 **	−0.009	0.531 **	0.475 **	1							
7. Health and medical care tasks	1.76	2.43	0.068 *	0.148 **	0.024	0.515 **	0.652 **	0.550 **	1						
8. Organization and coordination of health care tasks	1.54	2.21	0.076 *	0.162 **	0.020	0.485 **	0.651 **	0.513 **	0.784 **	1					
9. Acting as a substitute tasks	1.54	2.35	0.068 *	0.144 **	0.028	0.467 **	0.579 **	0.461 **	0.654 **	0.765 **	1				
10. Family-work conflict	1.30	0.68	−0.033	−0.048	0.184 **	0.103 **	0.146 **	0.155 **	0.173 **	0.239 **	0.173 **	1	(0.77)		
11. Emotional exhaustion	2.45	1.43	0.020	0.004	−0.014	0.065 *	0.060 *	0.119 **	0.100 **	0.104 **	0.084 **	0.359 **	1	(0.91)	
12. Presenteeism	1.11	0.40	0.072 *	−0.028	0.030	0.011	0.040	0.109 **	0.082 **	0.110 **	0.087 **	0.348 **	0.733 **	1	(0.84)
13. Absenteeism	1.34	1.28	0.211 **	0.049	0.119 **	0.077 *	0.165 **	0.125 **	0.272 **	0.298 **	0.294 **	0.230 **	0.112 **	0.214 **	1

* *p* < 0.05, ** *p* < 0.01, Alpha coefficients are reported within parentheses along the diagonal.

**Table 3:** The average hours spent per month on informal caregiving tasks for the participants in this study.

Tasks	Mean (Hours/Month)	SD
Housework	2.98	3.39
Personal care	1.69	2.59
Social and emotional support	3.75	3.60
Health and medical care	1.76	2.43
Organizing and coordinating healthcare	1.54	2.21
Acting as a substitute	1.54	2.35

## Data Availability

The data presented in this study are available upon request from the corresponding author. The data are not publicly available due to privacy restrictions.

## References

[1] World Health Organization (2022). Ageing and Health. https://www.who.int/news-room/fact-sheets/detail/ageing-and-health.

[2] United Nations (2019). World Population Prospects 2019.

[3] Retraite Québec (2016). Constats sur la Retraite au Québec.

[4] Latulipe D., St-Onge S., Gagné C., Ballesteros-Leiva F., Legault M.-È.B. (2017). Le proongement de la vie professionnelle des Québécois: Une nécessité pour la société, les travailleurs et les employeurs?. Retraite Et Société.

[5] Hopps M., Iadeluca L., McDonald M., Makinson G.T. (2017). The burden of family caregiving in the United States: Work productivity, health care resource utilization, and mental health among employed adults. J. Multidiscip. Healthc..

[6] Canadian Institute for Health Information (2022). Unpaid Caregiver Challenges and Supports. https://www.cihi.ca/en/dementia-in-canada/unpaid-caregiver-challenges-and-supports.

[7] Family Caregiver Alliance (2019). Caregiver Statistics: Demographics. https://www.caregivercalifornia.org/2021/05/06/caregiver-caregiving-statistics/.

[8] Statistics Canada (2015). Informal Caregiving for Seniors. https://www150.statcan.gc.ca/n1/pub/82-003-x/2012003/article/11694-eng.htm.

[9] L’appui (2016). L’appui Pour les Proches Aidants D’aînés. https://www.lappui.org/.

[10] CDC (2019). Caregiving for Family and Friends—A Public Health Issue. https://www.cdc.gov/aging/caregiving/caregiver-brief.html.

[11] Dumais L. (2018). In-Home and Caregiver Support—A Necessary Philanthropic Intervention?.

[12] Goverrnment of Canada EI Caregiving Benefits: Do You Qualify. https://www.canada.ca/en/services/benefits/ei/caregiving/eligibility.html.

[13] Evans C.J. (2004). Health and work productivity assessment: State of the art or state of flux?. J. Occup. Environ. Med..

[14] Johansson G., Lundberg I. (2004). Adjustment Latitude and Attendance Requirements as Determinants of Sickness Absence or Attendance. Empirical Tests of the Illness Flexibility Model. Soc. Sci. Med..

[15] Vézina M., Cloutier E., Stock S., Lippel K., Fortin E., Delisle A., St-Vincent M., Funes A., Duguay P., Vézina S. (2011). Enquête Québécoise sur les Conditions de Travail, D’emploi et de Santé et de Sécurité du Travail (EQCOTESST).

[16] Folger J. (2021). The Causes and Costs of Absenteeism. https://www.investopedia.com/articles/personal-finance/070513/causes-and-costs-absenteeism.asp.

[17] Zuba M., Schneider U. (2013). What helps working informal caregivers? The role of workplace characteristics in balancing work and adult-care responsibilities. J. Fam. Econ. Issues.

[18] Zacher H., Schulz H. (2015). Employees’ eldercare demands, strain, and perceived support. J. Manag. Psychol..

[19] Greenhaus J.H., Beutell N.J. (1985). Sources of Conflict between Work and Family Roles. Acad. Manag. Rev..

[20] Hobfoll S.E. (1989). Conservation of resources: A new attempt at conceptualizing stress. Am. Psychol..

[21] Maslach C., Jackson S.E. (1981). The measurement of experienced burnout. J. Organ. Behav..

[22] Feinberg L., Reinhard S.C., Houser A., Choula R. (2011). Valuing the Invaluable: 2011 Update, the Growing Contributions and Costs of Family Caregiving.

[23] The National Alliance for Caregiving (NARC) (2015). Caregiving in the U.S. 2015.

[24] Lecours C. (2015). Portrait des Proches Aidants et les Conséquences de Leurs Responsabilités D’aidant.

[25] Schulz R., Tompkins C.A. (2010). Informal Care. The Role of Human Factors in Home Health Care: Workshop Summary.

[26] Ferrara M., Langiano E., Di Brango T., Di Cioccio L., Bauco C., De Vito E. (2008). Prevalence of stress, anxiety and depression in with Alzheimer caregivers. Health Qual. Life Outcomes.

[27] Bevans M., Sternberg E.M. (2012). Caregiving burden, stress, and health effects among family caregivers of adult cancer patients. JAMA.

[28] Grandey A.A., Cropanzano R. (1999). The Conservation of Resources Model Applied to Work–Family Conflict and Strain. J. Vocat. Behav..

[29] Truzzi A., Valente L., Ulstein I., Engelhardt E., Laks J., Engedal K. (2012). Burnout in Familial Caregivers of Patients with Dementia.

[30] St-Armour N., Leverdure J., Devault A., Manseau S. (2005). La Difficulté de Concilier Travail-Famille: Ses Impacts sur la Santé Physique et Mentale des Familles Québécoises.

[31] Davis K.D., Gere J., Sliwinski M.J. (2017). Investigating the work–family conflict and health link: Repetitive thought as a mechanism. Stress Health.

[32] McDaniel K.R., Allen D.G. (2012). Working and Care-giving: The Impact on Caregiver Stress, Family-Work Conflict, and Burnout. J. Life Care Plan..

[33] Alarcon G.M. (2011). A meta-analysis of burnout with job demands, resources, and attitudes. J. Vocat. Behav..

[34] Bowling N.A., Alarcon G.M., Bragg C.B., Hartman M.J. (2015). A meta-analytic examination of the potential correlates and consequences of workload. Work Stress.

[35] Hobfoll S.E., Halbesleben J., Neveu J.-P., Westman M. (2018). Conservation of resources in the organizational context: The reality of resources and their consequences. Annu. Rev. Organ. Psychol. Organ. Behav..

[36] Mazanec S.R., Daly B.J., Douglas S.L., Lipson A.R. (2011). Work productivity and health of informal caregivers of persons with advanced cancer. Res. Nurs. Health.

[37] Demerouti E., Blanc P.M., Schaufeli W., Hox J. (2009). Present but sick: A three-wave study on job demands, presenteeism and burnout. Career Dev. Int..

[38] Clancy R.L., Fisher G.G., Daigle K.L., Henle C.A., McCarthy J., Fruhauf C.A. (2020). Eldercare and Work Among Informal Caregivers: A Multidisciplinary Review and Recommendations for Future Research. J. Bus. Psychol..

[39] Vignoli M., Guglielmi D., Bonfiglioli R., Violante F.S. (2016). How job demands affect absenteeism? The mediating role of work–family conflict and exhaustion. Int. Arch. Occup. Environ. Health.

[40] Schaufeli W.B., Bakker A.B., Van Rhenen W. (2009). How changes in job demands and resources predict burnout, work engagement, and sickness absenteeism. J. Organ. Behav. Int. J. Ind. Occup. Organ. Psychol. Behav..

[41] Roelen C., Van Hoffen M., Groothoff J., De Bruin J., Schaufeli W., Van Rhenen W. (2015). Can the Maslach Burnout Inventory and Utrecht Work Engagement Scale be used to screen for risk of long-term sickness absence?. Int. Arch. Occup. Environ. Health.

[42] Brislin R.W., Triandis H.C., Brislin R.W. (1980). Translation and content analysis of oral and written material. Handbook of Cross-Cultural Psychology.

[43] Turpin R.S., Ozminkowski R.J., Sharda C.E., Collins J.J., Berger M.L., Billotti G.M., Baase C.M., Olson M.J., Nicholson S. (2004). Reliability and validity of the Stanford Presenteeism Scale. J. Occup. Environ. Med..

[44] Gutek B.A., Searle S., Klepa L. (1991). Rational versus gender role explanations for work-family conflict. J. Appl. Psychol..

[45] Halinski M., Duxbury L., Stevenson M. (2020). Employed Caregivers’ Response to Family-Role Overload: The Role of Control-at-Home and Caregiver Type. J. Bus. Psychol..

[46] Gareth J., Daniela W., Trevor H., Robert T. (2013). An Introduction to Statistical Learning: With Applications in R..

[47] Anderson J.C., Gerbing D.W. (1988). Structural equation modeling in practice: A review and recommended two-step approach. Psychol. Bull..

[48] Preacher K.J., Hayes A.F. (2008). Asymptotic and resampling strategies for assessing and comparing indirect effects in multiple mediator models. Behav. Res. Methods.

[49] Hu L.-t., Bentler P.M. (1999). Cutoff criteria for fit indexes in covariance structure analysis: Conventional criteria versus new alternatives. Struct. Equ. Model..

[50] Podsakoff P.M., MacKenzie S.B., Lee J.Y., Podsakoff N.P. (2003). Common method biases in behavioral research: A critical review of the literature and recommended remedies. J. Appl. Psychol..

[51] Podsakoff P.M., MacKenzie S.B., Podsakoff N.P. (2012). Sources of Method Bias in Social Science Research and Recommendations on How to Control It. Annu. Rev. Psychol..

[52] Golden S., Nageswaran S. (2012). Caregiver Voices: Coordinating Care for Children with Complex Chronic Conditions. Clin. Pediatr..

[53] Boles M., Pelletier B., Lynch W. (2004). The Relationship Between Health Risks and Work Productivity. J. Occup. Environ. Med. Am. Coll. Occup. Environ. Med..

[54] Hemp P. (2004). Presenteeism: At work-but out of it. Harv. Bus. Rev..

[55] Karanika-Murray M., Biron C. (2019). The health-performance framework of presenteeism: Towards understanding an adaptive behaviour. Hum. Relat..

[56] Van den Broeck A., Ferris D.L., Chang C.-H., Rosen C.C. (2016). A Review of Self-Determination Theory’s Basic Psychological Needs at Work. J. Manag..

[57] Bakker A.B., Demerouti E., De Boer E., Schaufeli W.B. (2003). Job demands and job resources as predictors of absence duration and frequency. J. Vocat. Behav..

[58] Maxwell S.E., Cole D.A. (2007). Bias in cross-sectional analyses of longitudinal mediation. Psychol. Methods.

[59] Stratton S.J. (2021). Population Research: Convenience Sampling Strategies. Prehospital Disaster Med..

[60] Evans J.R., Mathur A. (2005). The value of online surveys. Internet Res..

